# Study on rehabilitation effect of continuous nursing on patients with dysphagia in acute cerebral infarction

**DOI:** 10.1097/MD.0000000000043141

**Published:** 2025-07-11

**Authors:** Chunyan Xu, Tingting Zhan

**Affiliations:** a Department of Neurology, Wuhan Fourth Hospital, Wuhan, Hubei Province, China; b Emergency Department, Wuhan Fourth Hospital, Wuhan, Hubei Province, China.

**Keywords:** acute cerebral infarction, continuous care, dysphagia, quality of life, rehabilitation outcomes

## Abstract

This study aims to investigate the impact of continuous care on the rehabilitation outcomes of patients with acute cerebral infarction accompanied by dysphagia. This retrospective study involved 119 patients with acute cerebral infarction and dysphagia admitted to Wuhan No. 4 Hospital from June 2022 to December 2023. Patients were divided into an experimental group (n = 56) receiving standard care plus continuous care, and a control group (n = 63) receiving standard care alone. Continuous care included follow-up calls, home visits, health education, and personalized rehabilitation. Outcomes assessed included swallowing function, Barthel Index, quality of life (SF-36, Stroke-Specific Quality of Life), medication adherence, mental health, complications, and readmission rates. In the experimental group, swallowing function scores increased from 3.1 ± 1.2 at baseline to 5.0 ± 0.7 at 6 months, while the control group increased from 3.4 ± 1.3 to 4.2 ± 0.8 (*P* < .05). The Barthel Index in the experimental group improved from 40.5 ± 10.3 to 85.3 ± 10.7 at 6 months, compared to an increase from 41.2 ± 9.8 to 78.5 ± 12.3 in the control group (*P* < .05). SF-36 scores in the experimental group rose from 60.5 ± 8.1 to 75.0 ± 6.5, while the control group increased from 61.2 ± 7.9 to 68.0 ± 6.8 (*P* < .05). The Stroke-Specific Quality of Life scores in the experimental group increased from 100.5 ± 15.3 to 130.2 ± 10.9 at 6 months, whereas the control group increased from 101.2 ± 14.9 to 120.5 ± 12.5 (*P* < .05). Medication adherence significantly improved in the experimental group, with the proportion of highly adherent patients increasing from 42.9% to 80.4% (*P* < .001), compared to an increase from 41.1% to 62.5% in the control group (*P* = .02). The incidence of aspiration pneumonia significantly lower than the control group (*P* = .02). The readmission rate was 14.3% in the experimental group, significantly lower than 30.4% in the control group (*P* = .002). Additionally, anxiety and depression scores in the experimental group were significantly reduced (*P* < .001). Continuous care significantly improves swallowing function, activities of daily living, and mental health in patients with acute cerebral infarction and dysphagia. It also reduces complications and readmission rates, demonstrating substantial clinical value.

## 1. Introduction

Stroke, particularly ischemic stroke, is one of the leading causes of mortality and disability among adults. It often results in a significant decline in patients’ quality of life, accompanied by various functional impairments, including dysphagia.^[1]^ Dysphagia is a common complication of acute ischemic stroke, affecting approximately 50% to 55% of patients.^[2]^ This swallowing difficulty can lead to severe complications such as aspiration pneumonia, malnutrition, and dehydration, which notably impede the rehabilitation process and increase the rate of hospital readmissions.^[3]^ Moreover, inadequate recovery of swallowing function may result in high disability rates, reducing patients’ ability to perform daily activities and imposing a heavy burden on both patients and their families.^[4]^

In recent years, advancements in medical technologies like thrombolysis and endovascular therapy have significantly reduced the mortality rate of stroke patients during the acute phase. However, the intense focus on acute treatment often leads to a neglect of secondary prevention and long-term functional rehabilitation, which are crucial for enhancing post-discharge rehabilitation outcomes and quality of life.^[5]^ Traditional nursing models primarily concentrate on inpatient care and basic discharge instructions, lacking continuity between hospital and home care. This gap results in suboptimal functional recovery, increased complications, and higher rates of hospital readmissions.^[6]^

Continuous care, as an emerging nursing model, aims to bridge the gap by providing ongoing, personalized care outside the hospital setting. This approach seeks to improve patients’ functional recovery and quality of life, thereby addressing the shortcomings of traditional nursing models.^[7]^ Previous studies have demonstrated that continuous care offers significant advantages in enhancing rehabilitation outcomes, quality of life, reducing complications, and lowering hospital readmission rates. For instance, the study by Dou et al indicated that continuous care, through sustained health education and follow-up, significantly reduced the incidence of complications such as aspiration pneumonia.^[8]^ Similarly, Bauer et al emphasized the positive impact of continuous care on improving patients’ mental health and reducing anxiety and depression.^[9]^

Despite some encouraging findings, evidence regarding the effectiveness of continuous care in stroke patients with dysphagia remains limited. Given the high prevalence of dysphagia and its associated complications in stroke patients, understanding the role of continuous care in this population is essential for improving rehabilitation outcomes and overall quality of life. Therefore, this study aims to evaluate the impact of continuous care on the rehabilitation outcomes of patients with acute ischemic stroke accompanied by dysphagia. The focus will be on swallowing function, activities of daily living (ADL), quality of life, medication adherence, as well as complications and hospital readmission rates. This study hypothesizes that, compared to standard care, continuous care will significantly enhance swallowing function, ADL, mental health, and medication adherence, while reducing complications and hospital readmission rates in patients with acute ischemic stroke and dysphagia.

## 2. Materials and methods

### 2.1. Study design and settings

This retrospective study was approved by the Ethics Committee of Wuhan No. 4 Hospital (Approval Number: WH4H-REC-2023-001, Approval Date: March 15, 2023). Informed consent was waived by the Ethics Committee due to the retrospective nature of the study, which involved the use of anonymized data from medical records. This retrospective study aimed to investigate the impact of continuous care on the rehabilitation outcomes of patients with acute cerebral infarction accompanied by dysphagia. A total of 119 patients who met the inclusion criteria for acute cerebral infarction and dysphagia were admitted to Wuhan No. 4 Hospital between June 2022 and December 2023. Based on whether they received continuous care, the patients were divided into 2 groups: the experimental group, which received standard care plus continuous care (n = 56), and the control group, which received standard care alone (n = 63).

*Inclusion criteria*: Diagnosis of acute ischemic cerebral infarction according to established diagnostic criteria, with disease onset not exceeding 14 days (the main references are the “Clinical Management Guidelines for Acute Ischemic Stroke of the Chinese Stroke Association^[10]^” and the “Management Guidelines for Dysphagia of the Chinese Stroke Association^[11]^”); confirmed presence of varying degrees of swallowing dysfunction following a dysphagia assessment; age between 40 and 85 years; stable medical condition without other complications that would prolong hospital stay; and provision of informed consent with complete follow-up data.

*Exclusion criteria*: History of chronic dysphagia or having undergone swallowing rehabilitation training previously; concurrent severe neurological disorders, such as Parkinson disease or Alzheimer disease, which could compromise the accuracy of swallowing function assessments; presence of severe cardiopulmonary insufficiency or liver and kidney failure, with an expected survival period of less than 6 months; history of mental illness or cognitive impairment, rendering the patient unable to cooperate with researchers for swallowing function assessments and rehabilitation training; pregnant or lactating women.

## 3. Comprehensive care process

### 3.1. Control group: standard care content

1. Patients in the control group received standard care and swallowing function training during hospitalization, along with basic discharge instructions upon discharge. The specific care components are as follows:
2. Medication use: instruct patients to take medications as prescribed at discharge, especially anticoagulants and antihypertensive drugs, paying attention to the timing, dosage, and potential side effects.
3. Follow-up appointments: advise patients to schedule regular follow-up visits, recommending a return to the hospital for reexamination one month after discharge.
4. Rehabilitation training recommendations: provide patients and their families with basic swallowing rehabilitation exercises, including mouth opening, mouth closing, and swallowing practices. Encourage patients to independently perform daily swallowing training at home.
5. Dietary guidance: recommend that patients consume primarily liquid or semiliquid foods, avoid hard or overly sticky foods, and adopt a small, frequent meal approach.
6. Precautions: remind patients to prevent the risk of aspiration and choking by avoiding talking or being distracted while eating.
7. Follow-up methods: conduct telephone follow-ups 1 month after discharge to primarily assess the patients’ basic rehabilitation status.

### 3.2. Experimental group: continuous care content^[12]^

The experimental group received all components of standard care plus the following distinctive interventions during the 12-week post-discharge period:

#### 3.2.1. Intensive follow-up system

*Structured schedule*: Added telephone follow-ups at weeks 1 and 8, complemented by home visits at weeks 4 and 12 (versus single phone follow-up in standard care).

*Standardized assessments*: Implemented validated scales (FOIS, MASA) during each contact to objectively track swallowing function progression.

#### 3.2.2. Dynamic rehabilitation protocol

*Personalized training plans*: Biweekly adjustments based on video-recorded swallowing performance and home visit observations.

*Stratified strategies*: Tailored exercises by dysphagia severity (e.g., transition protocols from liquids to semisolids for Rosenbek Scale levels V–VI).

#### 3.2.3. Multidimensional support

Three structured education sessions: delivered by SLP specialists covering:

Stroke pathophysiology and neuroplasticity principles.
Advanced home safety modifications (lighting, seating angles).
Complication prevention protocols (aspiration pneumonia signs).
Digital tools: provided tablet-based training videos with progress tracking functionality.

#### 3.2.4. Family-centered approach

*Caregiver certification program*: Required family members to demonstrate competency in:

Modified barium swallow supervision.
Heimlich maneuver execution.
Home environment audits: conducted systematic safety evaluations (e.g., optimal dining chair height).

### 3.3. Data collection

1. Basic information: age, gender, body mass index, history of diabetes, history of hypertension, smoking history, alcohol consumption history, education level, family support, length of hospital stay, NIHSS score.
2. Swallowing function assessment: Kubota Water Swallow Test.^[13]^ This test evaluates the normality of swallowing function by having patients drink water continuously while observing their swallowing responses. The scoring is based on whether the patient can complete the swallowing task smoothly and whether adverse reactions such as choking occur. Grade 0: normal, no choking, successfully completes drinking. Grade 1: mild dysphagia, slight choking, able to swallow but with discomfort. Grade 2: moderate dysphagia, significant choking, difficulty swallowing but partially completes the task. Grade 3: severe dysphagia, unable to swallow, immediate and obvious choking. Grade 4: critical swallowing impairment, completely unable to swallow, with evident aspiration.
3. Functional recovery: assessed using the Barthel Index^[14]^ to evaluate patients’ ability to perform ADL. Assessments were conducted at baseline, and during follow-ups at 1 month, 3 months, and 6 months to monitor the recovery of daily living functions. The Barthel Index ranges from 0 to 100 points, with higher scores indicating better functional recovery.
4. Quality of life: evaluated using the SF-36 (overall health status)^[15]^ and the stroke-specific quality of life (SS-QOL)^[16]^ scales. Measurements were taken at baseline, 1 month, 3 months, and 6 months to assess changes in quality of life over time. SF-36: Total score ranges from 0 to 100 points, with higher scores indicating better health status. SS-QOL: scores are used to assess the quality of life specific to stroke patients, with higher scores indicating better quality of life.
5. Medication adherence: assessed using the self-reported medication nonadherence measure (SRMN)^[17]^ to evaluate patients’ adherence to their medication regimen. The SRMN scale was used to assess whether patients were taking their medications as prescribed, specifically in terms of timing, dosage, and frequency. Assessments were conducted at baseline and at 6 months. The SRMN scale ranges from 0 to 8 points, with higher scores indicating poorer medication adherence. Lower scores reflect better adherence to the prescribed medication regimen.
6. Mental health: evaluated using the Generalized Anxiety Disorder-7 (GAD-7)^[18]^ and Patient Health Questionnaire-9 (PHQ-9)^[18]^ scales to assess patients’ anxiety and depression levels. The GAD-7 scale evaluates anxiety, while the PHQ-9 scale assesses depression. Both assessments were conducted at baseline and at 6 months to evaluate the psychological health status. Higher GAD-7 scores (ranging from 0–21) indicate more severe anxiety, while higher PHQ-9 scores (ranging from 0–27) indicate more severe depression.
7. Complications and readmission^[19]^: the primary complications observed included aspiration pneumonia, malnutrition/dehydration, pressure ulcers, deep vein thrombosis/pulmonary embolism, urinary tract infections, and psychological issues (anxiety/depression). The occurrence of each type of complication was recorded during the 6-month follow-up period.

Recorded the instances of patients being readmitted to the hospital for various reasons during the 6-month follow-up period. The readmission rates were statistically analyzed and compared between the experimental and control groups.

### 3.4. Statistical analysis

The calculation of the sample size was carried out through G*Power 3.1 software. In the absence of prior assumptions, we conducted a posterior sample size calculation. The main purpose of hypothesis testing is to evaluate the differences in the main outcome variables between the experimental group and the control group. Based on the medium effect size (Cohen d = 0.5), with the significance level set at α = 0.05 and the target statistical power at 0.80, it was calculated that the required sample size for each group was 56 patients.

Statistical analyses were performed using SPSS. Continuous variables were expressed as mean ± standard deviation (mean ± SD), while categorical variables were presented as frequencies and percentages. Between-group comparisons for baseline characteristics were performed using independent samples *t* tests or Mann–Whitney *U* tests for continuous data and chi-square tests (*χ*² tests) for categorical data.

To reduce confounding bias in baseline characteristics, propensity score matching was applied using 1:1 nearest-neighbor matching without replacement. Post-matching comparisons were reassessed to ensure group balance.

For outcome analysis, two-way repeated measures ANOVA was used to evaluate the main effects of time, group, and the interaction between time and group for primary and secondary outcome variables (e.g., swallowing function, Barthel Index, SF-36, SS-QOL, medication adherence, HADS scores). Where assumptions of sphericity were violated, Greenhouse-Geisser corrections were applied.

Additionally, the percentage change from baseline was calculated for both groups at each follow-up time point using the formula:


$$\begin{array}{l} Percentage\;change=\frac{Follow\text{-}up\;score-Baseline\;score}{Baseline\;score}\times 100\% \end{array}$$

For categorical outcomes such as complications and hospital readmission rates, chi-square tests were used. All statistical tests were two-tailed, and a *P*-value < .05 was considered statistically significant.

## 4. Results

### 4.1. Comparison of baseline characteristics between experimental and control groups before and after propensity score matching

Before propensity score matching, there were notable differences in several baseline characteristics between the experimental group (n = 56) and the control group (n = 63). For instance, the NIHSS score was significantly lower in the experimental group compared to the control group (9.5 ± 3.2 vs 10.8 ± 3.6, *P* = .03), indicating that patients in the experimental group had slightly milder conditions. Additionally, swallowing function scores exhibited a statistical difference (3.1 ± 1.2 vs 3.4 ± 1.3, *P* = .05), and the experimental group had a higher proportion of patients with good medication adherence (*P* = .04). No significant differences were observed between the 2 groups regarding other variables such as age, gender, diabetes mellitus, and length of hospital stay (*P* > .05), as shown in Table 1.

**Table 1:** Matching results of propensity score.

Variable	Pre-matching		Statistic value (*Z*/*χ*²)	*P* value	Post-matching		Statistic value (*Z*/*χ*²)	*P* value
	Experimental group (n = 56)	Control group (n = 63)			Experimental group (n = 56)	Control group (n = 56)		
Age (years, mean ± SD)	65.4 ± 7.2	66.1 ± 6.8	*Z* = 0.59	.55	65.4 ± 7.2	65.9 ± 7.0	*Z* = 0.35	.73
Gender (male/female, n)	32/24	38/25	*χ*²=0.12	.73	32/24	33/23	*χ*²=0.04	.84
NIHSS score (mean ± SD)	9.5 ± 3.2	10.8 ± 3.6	*Z* = 2.13	.03	9.5 ± 3.2	9.7 ± 3.4	*Z* = 0.23	.82
Swallowing dysfunction score (mean ± SD)	3.1 ± 1.2	3.4 ± 1.3	*Z* = 1.95	.05	3.1 ± 1.2	3.2 ± 1.1	*Z* = 0.35	.73
Diabetes (yes/no, n)	15/41	22/41	*χ*²=3.85	.05	15/41	16/40	*χ*²=0.05	.82
Hypertension (yes/no, n)	30/26	35/28	*χ*²=0.09	.76	30/26	31/25	*χ*²=0.04	.85
Hospital stay (days, mean ± SD)	12.4 ± 3.5	13.1 ± 4.1	*Z* = 1.01	.31	12.4 ± 3.5	12.8 ± 3.9	*Z* = 0.48	.63
Medication adherence (high/low, n)	50/6	52/11	*χ*²=4.12	.04	50/6	51/5	*χ*²=0.09	.76
Family support (good/poor, n)	44/12	40/23	*χ*²=3.13	.08	44/12	43/13	*χ*²=0.04	.84
Education level (high school+/below, n)	25/31	29/34	*χ*²=4.05	.04	25/31	26/30	*χ*²=0.05	.82
BMI (mean ± SD)	24.6 ± 3.2	25.1 ± 3.5	*Z* = 0.86	.39	24.6 ± 3.2	24.8 ± 3.4	*Z* = 0.31	.76
Smoking (yes/no, n)	20/36	18/45	*χ*²=0.65	.42	20/36	19/37	*χ*²=0.04	.84
Drinking (yes/no, n)	17/39	22/41	*χ*²=0.43	.51	17/39	18/38	*χ*²=0.02	.88

BMI = body mass index.

After propensity score matching, the baseline characteristics between the experimental and control groups were significantly balanced. There were no significant differences in age, gender, NIHSS score, swallowing function score, diabetes mellitus, hypertension, length of hospital stay, or medication adherence between the matched experimental and control groups (*P* > .05). This indicates that propensity score matching effectively eliminated confounding biases between the 2 groups, providing a more reliable foundation for subsequent outcome analyses.

### 4.2. Analysis of the impact of continuous care on swallowing function and functional recovery (Barthel Index)

The results, analyzed using two-way repeated measures ANOVA, showed no significant differences between the experimental and control groups at baseline (*P* > .05), as shown in Table 2. However, over time, the experimental group showed significantly greater improvements in swallowing function and functional recovery compared to the control group. At 1 month, the experimental group demonstrated an improving trend in swallowing function (*P* = .08). By 3 and 6 months, significant differences were observed (*P* < .05).

**Table 2:** Comparison of swallowing function and functional recovery (Barthel Index) between experimental and control groups using two-way ANOVA with interaction effects.

Variable	Group	Baseline	1 month (%Δ)	3 months (%Δ)	6 months (%Δ)	Group effect (*P*)	Time effect (*P*)	Group × Time interaction (*P*)
Swallowing function	Experimental	3.1 ± 1.2	3.8 ± 1.0 (+22.6%)	4.5 ± 0.9 (+45.2%)	5.0 ± 0.7 (+61.3%)	<.001	.001	.004
	Control	3.4 ± 1.3	3.6 ± 1.1 (+5.9%)	4.0 ± 1.0 (+17.6%)	4.2 ± 0.8 (+23.5%)			
Functional recovery (BI)	Experimental	40.5 ± 10.3	55.8 ± 12.1 (+37.8%)	70.6 ± 15.2 (+74.3%)	85.3 ± 10.7 (+110.6%)	<.001	.002	.003
	Control	41.2 ± 9.8	53.5 ± 11.5 **(+29.9%**)	65.7 ± 14.0 **(+59.5%**)	78.5 ± 12.3 **(+90.5%**)			

Bold values indicate statistical significance.

**Figure 1.**
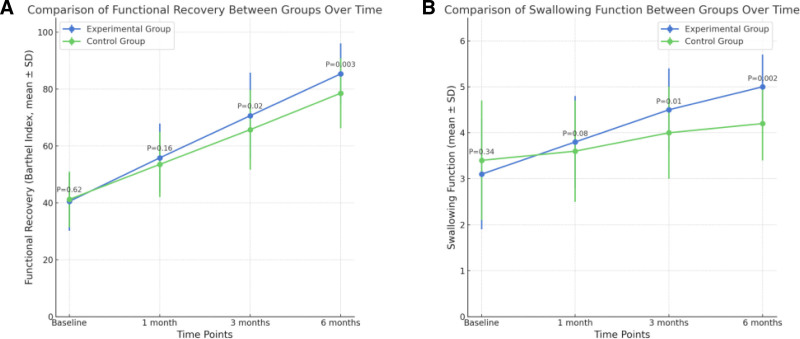
Comparison of (B) swallowing function and (A) functional recovery (Barthel Index) between experimental and control groups at different time points.

The group × time interaction was significant for both swallowing function (*P* = .004) and functional recovery (*P* = .003), indicating a greater improvement in the experimental group. The experimental group showed a more pronounced increase in swallowing function (+61.3%) and functional recovery (+110.6%) compared to the control group (+23.5% and +90.5%, respectively), as shown in Fig. 1.

These findings suggest that continuous care significantly improves swallowing function and functional recovery in patients with cerebral infarction and dysphagia.

### 4.3. Analysis of the impact of continuous care on overall health status (SF-36) and SS-QOL improvement

The results indicate that the experimental group showed significantly greater improvements in both SF-36 and SS-QOL scores compared to the control group. At baseline, there were no significant differences between the 2 groups in overall health status (SF-36) and SS-QOL (*P* > .05), as shown in Table 3. However, at the 3-month follow-up, the experimental group exhibited significant improvements in both scores (SF-36: *P* = .01; SS-QOL: *P* = .02). By the 6-month follow-up, the differences became even more pronounced, with the experimental group showing a significant increase in both SF-36 and SS-QOL scores (SF-36: *P* < .001; SS-QOL: *P* = .002).

**Table 3:** Comparison of SF-36 and SS-QOL scores between experimental and control groups at different time points.

Variable	Group	Baseline	1 month (%Δ)	3 months (%Δ)	6 months (%Δ)	Group effect (*P*)	Time effect (*P*)	Group × Time interaction (*P*)
SF-36 score	Experimental	60.5 ± 8.1	65.3 ± 7.5 (+7.9%)	70.2 ± 6.9 (+16.0%)	75.0 ± 6.5 (+23.9%)	<.001	.001	.004
	Control	61.2 ± 7.9	63.8 ± 7.2 (+4.2%)	66.5 ± 7.0 (+8.7%)	68.0 ± 6.8 (+11.1%)			
SS-QOL score	Experimental	100.5 ± 15.3	110.8 ± 13.2 (+10.2%)	120.3 ± 12.1 (+19.7%)	130.2 ± 10.9 (+29.6%)	<.001	.003	.005
	Control	101.2 ± 14.9	107.5 ± 12.8 (+6.2%)	115.0 ± 13.0 (+13.6%)	120.5 ± 12.5 (+19.1%)			

SS-QOL = stroke-specific quality of life.

Within-group comparisons revealed that the experimental group experienced significant enhancements in both quality of life measures from baseline to 6 months (*P* < .001 for both SF-36 and SS-QOL), whereas the control group also showed improvements, but to a lesser extent (*P* = .001 to *P* = .002). These findings suggest that continuous care interventions significantly improve patients’ overall health status and stroke-specific quality of life, with more substantial improvements seen in the experimental group.

### 4.4. Analysis of the impact of continuous care on medication adherence and mental health (anxiety and depression) improvement

During the 6-month follow-up, significant differences were observed between the experimental and control groups in medication adherence and mental health.

In terms of medication adherence, the experimental group showed a significant increase in the self-reported medication nonadherence measure score from 4.5 ± 1.5 at baseline to 6.2 ± 1.0 (+37.8%) (*P* < .001), while the control group showed a smaller increase from 4.7 ± 1.6 to 5.2 ± 1.4 (+10.6%) (*P* = .02), as shown in Table 4.

**Table 4:** Comparison of medication adherence (self-reported medication nonadherence measure), anxiety (GAD-7), and depression (PHQ-9) between experimental and control groups at baseline and 6 months.

Variable	Group	Baseline	6 months (%Δ)	Group effect (*P*)	Time effect (*P*)	Group × Time interaction (*P*)
Medication adherence (self-reported medication nonadherence measure)	Experimental	4.5 ± 1.5	6.2 ± 1.0 (+37.8%)	<.001	.001	.003
	Control	4.7 ± 1.6	5.2 ± 1.4 (+10.6%)			
Anxiety (GAD-7)	Experimental	13.5 ± 5.0	7.4 ± 3.5 (-45.0%)	<.001	.01	.02
	Control	13.8 ± 5.1	10.2 ± 4.3 (-26.1%)			
Depression (PHQ-9)	Experimental	15.0 ± 6.3	9.2 ± 4.0 (-38.7%)	<.001	.02	.02
	Control	14.8 ± 6.5	12.0 ± 5.5 (-18.9%)			

GAD-7 = Generalized Anxiety Disorder-7, PHQ-9 = Patient Health Questionnaire-9.

Regarding mental health, the experimental group exhibited significant reductions in anxiety (GAD-7) and depression (PHQ-9) scores. Anxiety scores decreased from 13.5 ± 5.0 to 7.4 ± 3.5 (‐45.0%) (*P* < .001), and depression scores decreased from 15.0 ± 6.3 to 9.2 ± 4.0 (‐38.7%) (*P* < .001). In contrast, the control group showed smaller reductions in anxiety (‐26.1%) and depression (‐18.9%) (*P* = .03 and *P* = .02, respectively).

These findings indicate that continuous care significantly improves medication adherence and reduces anxiety and depression compared to standard care.

### 4.5. Analysis of the impact of continuous care on complication incidence and hospital readmission rates

During the 6-month follow-up period, there were significant differences in the incidence of complications and hospital readmission rates between the experimental and control groups. The incidence of aspiration pneumonia in the experimental group was 8.9%, significantly lower than the 21.4% observed in the control group (*P* = .02). Similarly, the incidence of pressure ulcers in the experimental group was 1.8%, markedly lower than the 8.9% in the control group (*P* = .04), as shown in Table 5. Other complications, such as malnutrition, deep vein thrombosis, urinary tract infections, and psychological issues (anxiety/depression), occurred at lower rates in the experimental group compared to the control group, although these differences did not reach statistical significance (*P* > .05).

**Table 5:** Comparison of complication rates and readmission rates between experimental and control groups at 6 months.

Complication type/readmission	Experimental group (n = 56, 6 months)	Control group (n = 56, 6 months)	*X*^2^ value	*P* value
Aspiration pneumonia	5/56 (8.9%)	12/56 (21.4%)	*Z* = 2.25	*P* = .02
Malnutrition/dehydration	2/56 (3.6%)	6/56 (10.7%)	*Z* = 1.50	*P* = .13
pressure ulcer	1/56 (1.8%)	5/56 (8.9%)	*Z* = 2.05	*P* = .04
DVT/PE	1/56 (1.8%)	4/56 (7.1%)	*Z* = 1.80	*P* = .07
UTI	2/56 (3.6%)	4/56 (7.1%)	*Z* = 0.65	*P* = .51
Psychological issues (anxiety/depression)	1/56 (1.8%)	4/56 (7.1%)	*Z* = 1.80	*P* = .07
Overall complication rate	10/56 (17.9%)	18/56 (32.1%)	*Z* = 2.45	*P* = .01
Readmission rate	8/56 (14.3%)	17/56 (30.4%)	*Z* = 3.15	*P* = .002

DVT/PE = deep vein thrombosis/pulmonary embolism, UTI = urinary tract infections.

The overall complication rate in the experimental group was 17.9%, significantly lower than the 32.1% observed in the control group (*P* = .01). Additionally, the hospital readmission rate in the experimental group was 14.3%, which was significantly lower than the 30.4% in the control group (*P* = .002). These results indicate that continuous care has a significant advantage in reducing the incidence of complications and lowering hospital readmission rates.

## 5. Discussion

This study compared the effects of continuous care versus standard care in patients with acute cerebral infarction accompanied by dysphagia, exploring its advantages in promoting the recovery of swallowing function, improving ADL and mental health, enhancing medication adherence, and reducing complications and readmission rates.^[20]^ Acute cerebral infarction is one of the leading causes of mortality and disability among adults, and dysphagia is a common complication that significantly impacts quality of life and rehabilitation outcomes. Traditional care primarily focuses on inpatient nursing and basic discharge instructions, lacking systematic and continuous interventions. In recent years, continuous care has emerged as a novel nursing model, gaining attention for improving patients’ functional recovery and quality of life through outpatient continuous care services.^[21]^ The results of this study indicate that continuous care significantly promotes patient rehabilitation, especially in areas such as swallowing function, Barthel Index, overall health status (SF-36), SS-QOL, and medication adherence, thereby supporting its clinical value in stroke rehabilitation.

Firstly, the significant promotive effect of continuous care on the recovery of swallowing function is noteworthy. The study results demonstrate that the experimental group showed markedly better swallowing function than the control group at both the 3-month and 6-month post-discharge follow-ups. This indicates that the interventions associated with continuous care (such as telephone follow-ups, home visits, and personalized swallowing rehabilitation training) play a crucial role in the rehabilitation of patients’ swallowing functions. Continuous care enables more timely identification and intervention of issues during the rehabilitation process through regular monitoring and personalized adjustments to the rehabilitation training programs, thereby accelerating the recovery of swallowing function.

Secondly, this study found that continuous care also has significant advantages in promoting the recovery of patients’ ADL. Changes in the Barthel Index indicated that functional recovery in the experimental group at 6 months was significantly superior to that of the control group, suggesting that continuous care can better assist patients in regaining their ability to perform daily self-care activities. Through on-site guidance and personalized rehabilitation plan adjustments during home visits, continuous care ensured that rehabilitation training continued effectively in the home environment.

Furthermore, continuous care demonstrated significant effects on improving patients’ overall health status and quality of life. At the 3-month and 6-month follow-ups, the experimental group significantly outperformed the control group in both SF-36 and SS-QOL scores, indicating that continuous care not only focuses on patients’ physical rehabilitation but also provides comprehensive support for psychological and social functions. Interventions such as health education lectures and psychological support played important roles in enhancing patients’ and their families’ understanding and cooperation with rehabilitation, thereby improving the patients’ overall quality of life.^[22]^

Medication adherence and mental health were also important evaluation indicators in this study. Patients in the experimental group showed a significant improvement in medication adherence and a notable reduction in anxiety and depression symptoms, indicating that continuous care plays a crucial role in enhancing patients’ initiative and compliance with medication regimens. Continuous care, through telephone follow-ups and home visits, timely assessed patients’ medication adherence and provided personalized medication guidance based on patients’ needs, effectively improving medication adherence.^[23]^ The improvement in mental health was primarily attributed to the psychological support within continuous care. Regular psychological communication helped patients alleviate anxiety and depression, thereby enhancing their confidence in the rehabilitation process.

Finally, continuous care also demonstrated significant effects in reducing the incidence of complications and lowering hospital readmission rates. The incidence of aspiration pneumonia and pressure ulcers in the experimental group was significantly lower than that in the control group, and the overall complication rate and hospital readmission rate were also markedly reduced. This indicates that continuous care, through home visits and health education, can timely identify and intervene with potential risk factors, effectively preventing the occurrence of complications. Specifically for patients with dysphagia, continuous care effectively reduced the risk of aspiration and the incidence of aspiration pneumonia by guiding proper dietary practices and swallowing training.

The results of this study are consistent with previous research findings. Existing studies have shown that continuous care has significant advantages in improving patients’ quality of life, promoting functional recovery, and reducing complications and hospital readmission rates. This study further validates the effectiveness of continuous care in patients with dysphagia and demonstrates its positive impact on medication adherence and mental health. Similar to the study by Konof et al, this research found that continuous care significantly reduced the incidence of complications such as aspiration pneumonia, which may be related to the ongoing health education and home visits inherent in continuous care.^[24]^ Additionally, this study emphasized the positive role of continuous care in improving mental health, aligning with Bauer et al’s findings on the role of continuous care in alleviating patients’ anxiety and depression.^[25]^

Overall, the combined results of this study and previous research indicate that continuous care, through systematic and personalized interventions, can effectively improve patients’ swallowing function, ADL, mental health status, and reduce complications and readmission rates. These findings provide stronger evidence supporting the application of continuous care in the rehabilitation of patients with acute cerebral infarction and dysphagia.

The results of this study support continuous care as a crucial intervention for the rehabilitation of patients with acute cerebral infarction and dysphagia. Compared to standard care, continuous care has significant advantages in restoring swallowing function, improving quality of life, enhancing medication adherence, and preventing complications. This care model, through personalized, systematic, and continuous interventions, not only enhances patient rehabilitation outcomes but also alleviates the pressure on medical resources, demonstrating high clinical applicability.

However, this study also has several limitations. Firstly, it is a single-center retrospective study with a limited sample size, including only patients from Wuhan No. 4 Hospital. This may introduce selection bias and limit the external validity and generalizability of the results. Future studies should consider multicenter, large-sample designs to enhance the representativeness of the sample and improve the generalizability of the conclusions. Secondly, the implementation of continuous care in this study relied on the experience and capabilities of nursing staff, which may have varied. Differences in the skill levels and adherence to the care protocol among different nurses could lead to inconsistent care outcomes, potentially increasing the uncertainty of the results. Further research should standardize continuous care protocols to minimize variability in implementation. Additionally, the adherence to continuous care in this study depended on the active participation of patients and their families. However, adherence can be influenced by various factors, such as cultural, social, and economic conditions, which were not fully assessed or controlled in this study. These factors may have contributed to variability in outcomes and should be considered in future research. Lastly, the follow-up period of 6 months is relatively short to evaluate the long-term effects of continuous care on rehabilitation outcomes. The benefits observed in this study may not fully reflect the long-term impact of continuous care. Future studies with extended follow-up periods could better assess the sustained effects of continuous care on long-term rehabilitation outcomes. To address these limitations, a prospective study is planned, which will involve a larger, multicenter sample and a longer follow-up period. This study will also standardize the continuous care protocol, assess adherence factors more comprehensively, and evaluate the long-term effects of continuous care on rehabilitation outcomes in a more controlled setting.

## 6. Conclusion

In summary, the application of continuous care in patients with acute cerebral infarction accompanied by dysphagia demonstrates significant advantages. It effectively improves patients’ swallowing function, ADL, and mental health status, while reducing complications and hospital readmission rates. Future efforts should further enhance the standardization and systematization of continuous care, promoting this nursing model to better serve stroke patients, improve their quality of life, and facilitate comprehensive rehabilitation.

## Author contributions

**Conceptualization:** Chunyan Xu, Tingting Zhan.

**Data curation:** Chunyan Xu, Tingting Zhan.

**Formal analysis:** Chunyan Xu, Tingting Zhan.

**Investigation:** Chunyan Xu, Tingting Zhan.

**Methodology:** Chunyan Xu, Tingting Zhan.

**Validation:** Chunyan Xu.

**Visualization:** Chunyan Xu.

**Writing – original draft:** Chunyan Xu, Tingting Zhan.

**Writing – review & editing:** Chunyan Xu, Tingting Zhan.
